# Rebuilding strength: surgical intervention and rehabilitation for bilateral spontaneous quadriceps tendon rupture—a case report

**DOI:** 10.3389/fsurg.2024.1430774

**Published:** 2024-07-18

**Authors:** Huaize Dong, Jin Yang, Hao Yu, Jinsong Zhu, Jibin Yang

**Affiliations:** Department of Orthopedic Surgery, Affiliated Hospital of Zunyi Medical University, Guizhou, China

**Keywords:** bilateral, quadriceps tendon rupture, spontaneous, surgical repair, rehabilitation

## Abstract

The quadriceps tendon, crucial for body movement, is among the body's strongest tendons. Factors like diabetes or hormone use can weaken it, making even minor trauma potentially causing rupture. Bilateral spontaneous quadriceps tendon rupture, where both tendons tear simultaneously, is rare. Prompt diagnosis and treatment are crucial. We present a case of a 44-year-old woman who experienced bilateral rupture after falling while doing chores. She had immediate pain and limited knee movement. Diagnosis via physical examination and CT/MRI scans confirmed the rupture. Surgical repair followed by rehabilitation led to significant pain reduction and improved function within two months. Overall, her postoperative outcome was satisfactory. This study underscores the importance of clear diagnosis, timely surgery, and thorough rehabilitation for optimal patient recovery from bilateral quadriceps tendon rupture.

## Introduction

The simultaneous rupture of the quadriceps femoris tendon is an exceptionally rare occurrence, given its status as one of the most robust tendons in the body. Studies suggest a higher incidence of quadriceps tendon rupture in men compared to women (1). Chronic underlying health conditions, such as diabetes (2), nephrotic syndrome (3), and prolonged hormone use (4), have been identified as common predisposing factors for quadriceps tendon rupture. Previous reports indicate that patients with these chronic conditions often experience widespread tendon degeneration, leading to decreased tendon mass and strength. Consequently, even minor trauma can precipitate unilateral or bilateral quadriceps tendon rupture (2). This underscores the importance of recognizing and managing underlying health conditions to mitigate the risk of tendon injuries.

Furthermore, spontaneous bilateral simultaneous rupture of the quadriceps tendon has been seldom reported, rendering it a relatively uncommon condition (5). Given its rarity, clinicians may encounter challenges in accurately diagnosing this condition, leading to potential misdiagnosis or missed diagnosis when distinguishing it from other disorders. Inaccurate initial diagnosis can result in delays in treatment initiation, thereby impeding therapeutic progress and ultimately impacting treatment outcomes. As one of the key tendons in the human body, the quadriceps femoris tendon plays a crucial role in the motor system. By enhancing awareness of spontaneous bilateral quadriceps tendon rupture, early and accurate diagnosis in clinical practice can be achieved, allowing for prompt surgical intervention to alleviate the patient's pain as soon as possible. Besides this, in addition to surgical intervention, postoperative rehabilitation assumes a pivotal role in managing this condition effectively, for patients, the goal of surgery is to repair and reconstruct the ruptured quadriceps tendons. However, the ultimate objective of diagnosing and treating this condition is to enable patients to achieve better functionality in their daily lives, return to normal activities, and resume sports. Therefore, postoperative rehabilitation and functional recovery play a role as crucial as the surgery itself in the overall treatment process (6). By emphasizing both surgical treatment and comprehensive rehabilitation, clinicians can optimize patient outcomes and facilitate a smoother recovery process.

Therefore, this article aims to report a case of spontaneous bilateral quadriceps tendon rupture in a female patient and to describe the treatment process. Additionally, it includes a literature review on quadriceps tendon rupture. By doing so, the article seeks to enhance clinical doctors' awareness of this condition, enabling them to diagnose it quickly and accurately and perform early surgical intervention, thus maximizing patient benefits by alleviating their pain. Moreover, by reflecting on and summarizing the rarity of spontaneous bilateral quadriceps tendon rupture, researchers can explore not only the various risk factors and triggers but also genetic aspects. This ongoing investigation into the disease's etiological mechanisms will provide valuable insights for its prevention and intervention.

## Case report

In this case report, we document the presentation of a 44-year-old woman who presented to our hospital with bilateral lower limb pain and restricted mobility following a fall. The patient reported accidentally kneeling on the ground while engaged in household chores at home approximately 10 h prior to admission. Upon impact, the patellae of both knee joints made contact with the ground first, resulting in immediate bilateral knee pain and limited mobility. Subsequently, the patient was transported to a local hospital by family members. Upon physical examination and MRI evaluation, bilateral quadriceps tendon rupture was diagnosed, prompting referral to our facility for further surgical intervention. Upon admission, our physical examination revealed mild bilateral knee joint swelling, tenderness, a positive floating patella test, and positive patella apprehension test. The patient's VAS pain score for both lower limbs is 8. Due to the pain, muscle strength in both lower limbs is rated as Grade 1. Extensive medical history inquiry revealed that the patient was not in the perimenopausal stage and had a history of regular physical examinations. Importantly, there were no underlying medical conditions such as hypertension, diabetes, coronary artery disease, autoimmune disorders, renal impairment, hyperthyroidism, hypothyroidism, or systemic lupus erythematosus. Furthermore, there was no history of prolonged hormone use.

**Figure 1:**
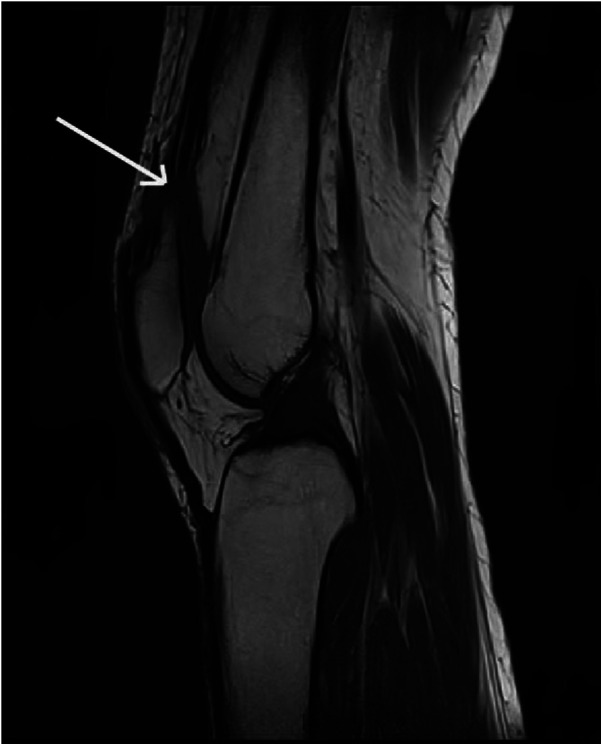
Preoperative MRI of the knee showed rupture of the quadriceps femoris tendon.

Following the meticulous examination of x-ray and MRI images of the bilateral knee joints, notable findings were observed. X-ray examination revealed a significant downward displacement of the upper pole of the bilateral patellae compared to normal knee joint anatomy. Moreover, MRI imaging depicted evident bilateral quadriceps femoris muscle ruptures, accompanied by surrounding soft tissue swelling, hematoma formation, and fluid accumulation (Figure 1). Subsequent laboratory investigations yielded reassuring results, indicating normal renal function, liver function, calcium and phosphorus levels, blood glucose, and other pertinent parameters. Furthermore, the patient's BMI value, calculated based on her height and weight, was determined to be 19.7, falling within the normal range.

**Figure 2:**
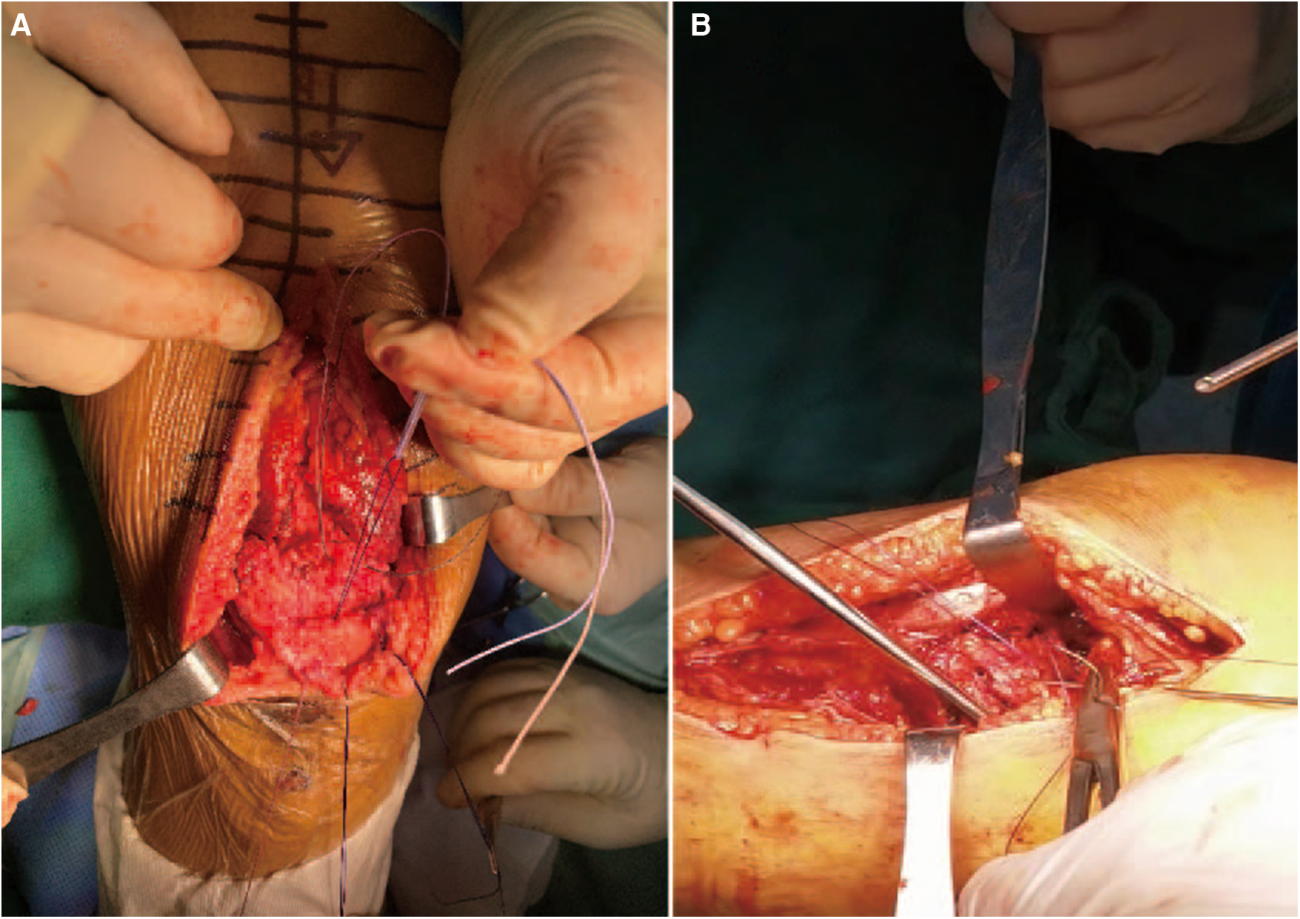
(**A**, **B**) Intraoperative absorbable suture was knitted and knotted through the patellar bone tunnel.

**Figure 3:**
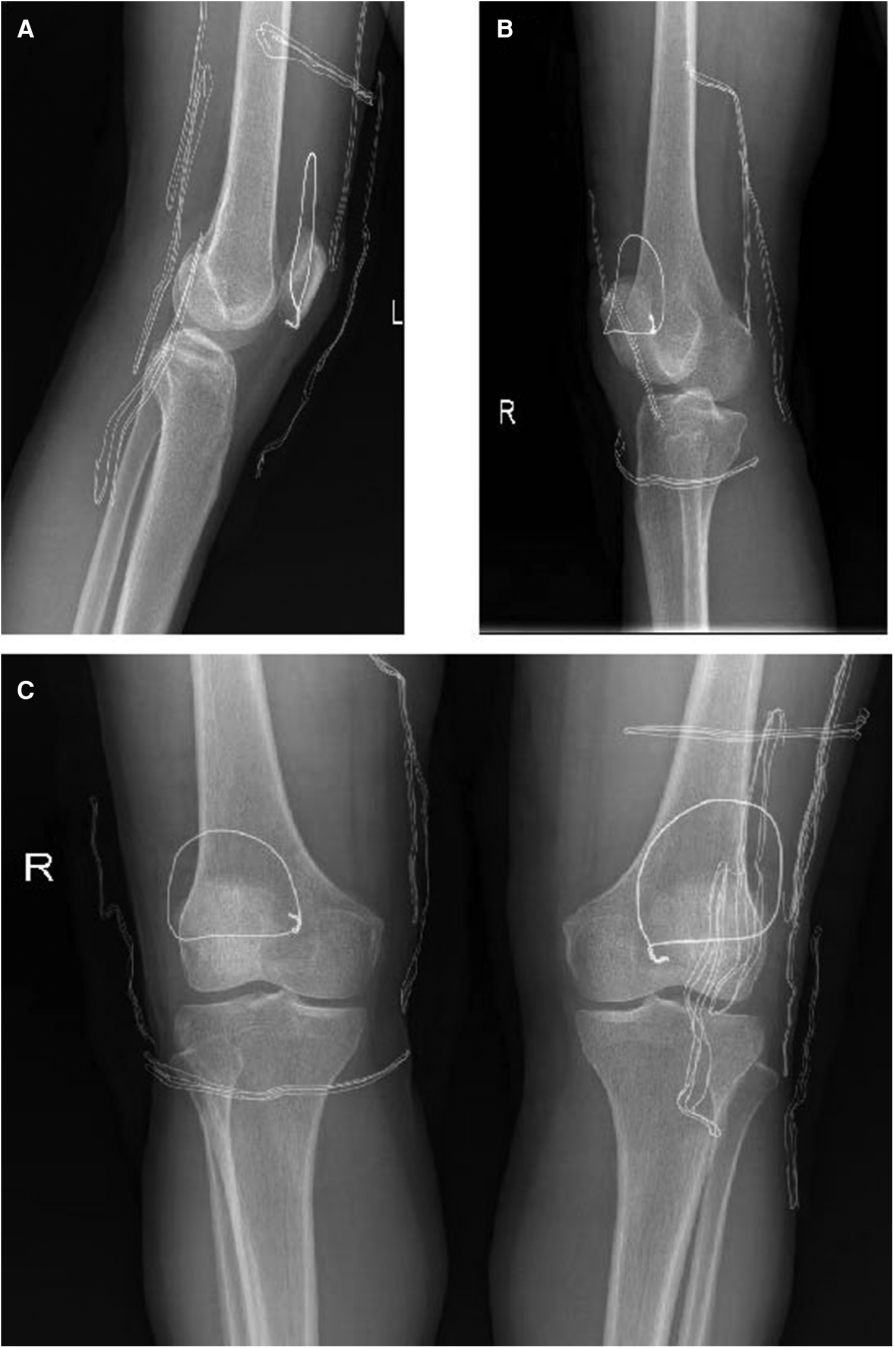
On the first day after operation, x-ray films of both knee joints were re-examined. (**A**) Lateral x-ray of the left knee; (**B**) Lateral x-ray of the right knee; (**C**) Lateral x-ray of the left knee.

Upon admission to the hospital, the patient received standard edema reduction therapy. Following subsidence of swelling in the affected region, bilateral quadriceps tendon repair was performed under general anesthesia. The surgical procedure commenced with routine disinfection of the bilateral knee joints, followed by exsanguination and tourniquet application. A 15 cm anteromedial incision was made on the left knee, successively dissecting through the skin, subcutaneous tissue, and fascia using an electric knife, with electrocoagulation employed for hemostasis. Upon identification of the ruptured quadriceps femoris tendon, characterized by rounded and hardened broken ends and significant fat infiltration between ligaments, diseased tendon tissue was excised. Suturing of the quadriceps femoris tendon stump was performed using two braided sutures. The upper pole of the patella was exposed, and a Kirschner wire was inserted, with sutures passed through a bone tunnel and knotted (Figure 2). Medial retinaculum and joint capsule tears were sutured using absorbable sutures, followed by knee joint movement to 70° to assess tendon integrity. A 1 cm steel wire was then looped around the mid-pole of the patella and the lower pole of the patella to alleviate tension. Knee joint movement was reassessed, ensuring motion exceeding 90° without impingement. Tourniquet release facilitated complete cessation of bleeding, with local hemostatic agents applied to reinforce hemostasis. Layered closure of the joint capsule without knotting sutures was performed, followed by pressure bandaging. The right knee joint underwent similar repair procedures as the left. Postoperatively, the patient received edema reduction, analgesia, anticoagulation, and external bracing. On the first postoperative day, the knee joints were re-examined in the anterolateral position, confirming the secure fixation of steel wires (Figure 3). On the first day after surgery, the patient's VAS pain score decreased to 5, but the muscle strength in both lower limbs remained at Grade 1 due to the pain.

Postoperatively, the patient was transferred to the rehabilitation department to undergo a comprehensive rehabilitation program. For the first month, exercises were performed with the use of a medically adjustable external fixation brace. The rehabilitation plan is as follows:

(1) Ankle Pump Exercises: Dorsiflex the foot with maximum force for 5 s, then plantarflex for 5 s. Perform 500–1,000 repetitions daily.
(2) Quadriceps Contractions: Contract the front thigh muscles for 5 s, then relax for 2 s. Perform 200 repetitions daily.
(3) Active Straight Leg Raises: With the knee joint in the brace, raise the leg straight off the bed to a 15° angle, holding until fatigued. Perform 4 times daily.
(4) Passive Knee Flexion: Achieve 90° of knee flexion within the first month postoperatively.
(5) Active Knee Flexion: Beginning in the second month, actively flex the knee to an angle greater than 90° but not exceeding 120°.

**Figure 4:**
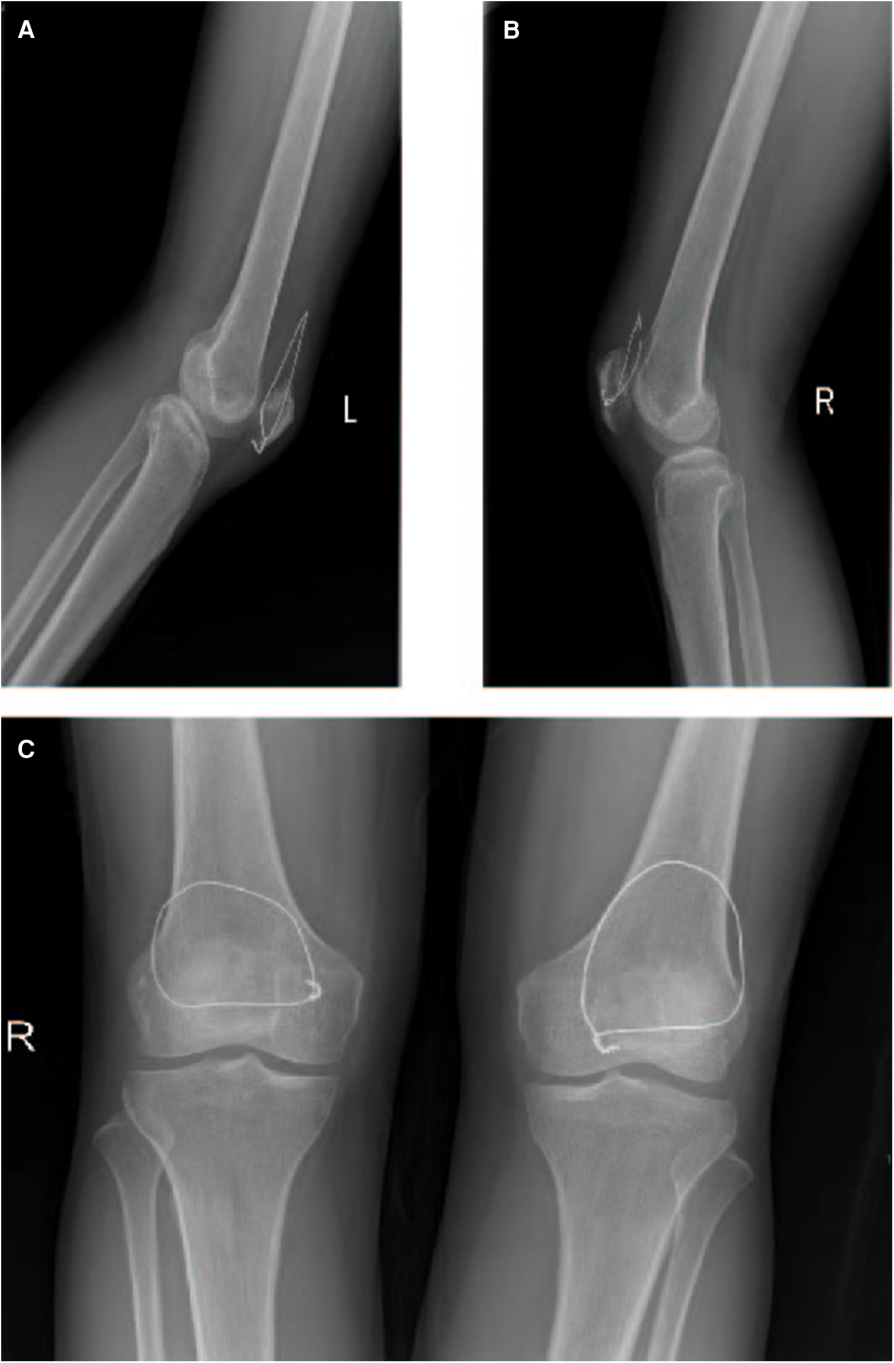
X-ray films of both knee joints were reexamined 3 months after operation. (**A**) Lateral x-ray of the left knee; (**B**) Lateral x-ray of the right knee; (**C**) Lateral x-ray of the left knee.

During the third-month follow-up post-surgery, x-ray examination of the patient's knee joints revealed satisfactory outcomes. The patellar position remained stable, and the absorbable sutures had completely dissolved (Figure 4). One month post-surgery, the patient's VAS pain score for both knees was 3. In the second month post-surgery, the pain score for both knees was 1, and by the third month, it had reduced to 0. Regarding knee range of motion, in the first month post-surgery, the passive range of motion in both knees was 0°–110°. In the second month, passive range of motion reached 120°, and active range of motion reached 90°. By the third month, active range of motion in both knees was 110°–120°. In terms of quadriceps muscle strength, by the end of the first month post-surgery, muscle strength in both lower limbs had recovered to Grade 4. By the end of the second month, muscle strength had recovered to Grade 5, allowing the patient to walk while bearing their own weight. By the third month, muscle strength in both lower limbs was assessed as Grade 5, enabling the patient to walk while bearing their own weight plus an additional 5–6 kg. Considering the comprehensive assessment of these conditions, we concluded that the patient had achieved favorable postoperative recovery from bilateral quadriceps tendon rupture, and the patient expressed satisfaction with the surgical outcome.

## Discussion

The quadriceps femoris tendon constitutes a crucial component of the human motor system and ranks among the strongest tendons in the human body (7). Research indicates that when quadriceps tendon fibers endure more than a 50% break under physiological loads, they become susceptible to rupture (1). Consequently, traumatic incidents causing quadriceps tendon rupture are typically forceful, or alternatively, diminished quadriceps tendon strength due to systemic conditions can predispose individuals to rupture even under mild stress (8). Conversely, spontaneous and bilateral quadriceps tendon ruptures are rare occurrences, as evidenced by previous case reports. Hence, enhancing comprehension of such uncommon conditions is imperative for clinicians. This knowledge equips clinicians with heightened vigilance during diagnosis and treatment, thereby mitigating the risk of misdiagnosis and oversight when encountering patients presenting with these conditions.

Considering the risk factors associated with quadriceps tendon rupture, numerous cases have been documented wherein the rupture of the quadriceps tendon correlates with underlying systemic conditions. Current research suggests that quadriceps tendon rupture can manifest as a secondary consequence of the progression of other systemic diseases. For instance, patients with nephrotic syndrome undergoing long-term hemodialysis are predisposed to quadriceps tendon rupture (9, 10). Scholars attribute this phenomenon to abnormal calcium and phosphorus metabolism in nephrotic syndrome patients, resulting in diminished quadriceps tendon strength and susceptibility to rupture under minimal force. Similarly, Stephany H et al. (11) reported a case of spontaneous bilateral quadriceps tendon rupture associated with severe vitamin D deficiency, underscoring the importance of calcium and phosphorus metabolism in tendon strength. Furthermore, diabetic patients have been reported to exhibit an increased risk of quadriceps tendon rupture (4), potentially linked to long-term elevated blood sugar levels impacting local metabolism, leading to tendon fragility and weakened strength. Some scholars argue that peripheral vascular damage caused by diabetes mellitus leads to poor blood supply to the quadriceps tendon, ultimately reducing its strength. As a country with a high prevalence of diabetes, China faces complications such as nephropathy (12), eye diseases, and diabetic foot syndrome. While quadriceps tendon rupture is considered less severe compared to these complications, its occurrence still significantly impacts the patient's quality of life. Therefore, enhancing the focus on diabetes is crucial. Long-term drug usage is also recognized as a risk factor for quadriceps tendon rupture. Chu et al. (13) reported a case of spontaneous quadriceps tendon rupture in a patient on prolonged norfloxacin therapy, positing that the drug influenced tendon strength via calcium and phosphorus metabolism. Moreover, long-term fluoroquinolone use has been associated with an elevated risk of quadriceps tendon rupture (14). Another noteworthy risk factor is hyperparathyroidism, a condition affecting calcium and phosphorus metabolism regulation. Chen et al. (8) identified a case of quadriceps femoris tendon rupture associated with hyperparathyroidism. Additionally, rare instances of non-traumatic bilateral quadriceps tendon rupture caused by patellar bone spurs have been reported (15). Continuous stimulation of the quadriceps tendon by hyperplastic patellar bone spurs leads to tendinopathy, weakening tendon strength and eventually culminating in rupture. Moreover, bilateral quadriceps tendon rupture has been observed following total knee replacement surgery (16). Although quadriceps tendon rupture is not commonly associated with knee replacement surgery, patients undergoing such procedures may experience tendon injuries, prosthetic discomfort, or over-resection of the patella, which could contribute to this complication.

The intricate interplay of calcium and phosphorus metabolism levels profoundly influences the strength of the quadriceps femoris tendon. Within the human body, multiple organs intricately regulate calcium and phosphorus metabolism, including the kidneys, intestinal tract, parathyroid glands, and bones. Decreased serum calcium levels stimulate parathyroid hormone (PTH) secretion, leading to the release of free calcium from bone stores and the induction of 1,25-(OH)-vitD3 secretion by the kidneys, thereby enhancing calcium absorption in the intestine (6). Consequently, severe damage to these organ systems can disrupt hormone balance, directly impacting calcium and phosphorus metabolism and exerting a chronic effect on tendon integrity. In light of this understanding, it prompts us to contemplate whether spontaneous quadriceps tendon rupture in an otherwise unidentified patient could serve as an early indicator of underlying systemic disease within the aforementioned systems. Wani et al. (17) elucidated that spontaneous quadriceps tendon rupture may serve as the initial presentation of chronic kidney disease. Similarly, Hamza et al. (18) demonstrated that spontaneous bilateral quadriceps tendon rupture can signal the presence of parathyroid carcinoma. These observations suggest a potential association between spontaneous quadriceps tendon rupture and the slow progression of systemic diseases, where tendon rupture manifests prior to the definitive diagnosis of the underlying condition. Moreover, this association may also be attributed to a high degree of genetic overlap between genes regulating quadriceps tendon-related phenotypes and those influencing other systemic diseases, warranting further investigation. Nevertheless, these insights highlight the importance of vigilant screening for related systemic diseases when encountering patients with quadriceps tendon rupture of unclear etiology in clinical practice. Timely detection and intervention can facilitate prompt treatment, potentially mitigating the progression and complications of underlying systemic conditions.

Surgical intervention is the preferred approach for the majority of patients with quadriceps femoris tendon rupture, playing a pivotal role in determining patient prognosis and recovery. The fundamental principle of surgical treatment involves the reconstruction and repair of the quadriceps femoris tendon, with the specific method tailored to each patient's unique condition. There is some controversy regarding whether surgery is always necessary for such patients. Some scholars believe that immobilizing the affected limb can allow scar healing of the quadriceps tendon, achieving similar repair effects while avoiding the potential decrease in tendon strength and the risk of infection associated with open surgery (19). Others argue that early surgical intervention, followed by prompt rehabilitation, is essential for patients to recover quickly and benefit from the treatment. This article acknowledges these differing viewpoints but emphasizes that the anatomical structure of the quadriceps tendon leads to poor blood supply, which can increase the risk of postoperative infection with open surgery. However, we can mitigate the risk of infection through thorough preoperative skin cleaning, strict aseptic techniques during surgery, careful handling to avoid vascular damage, and the timely prophylactic use of antibiotics before and after surgery. Current results show that the patient did not experience any infections and instead achieved good functional recovery of both lower limbs through early rehabilitation exercises post-surgery. Therefore, we recommend early surgery and prompt rehabilitation for such patients to achieve better outcomes. One commonly employed surgical technique is the modified Mason-Allen suture method, as utilized in the case of the patient discussed in this article. However, it's essential to acknowledge that various surgical modalities exist for quadriceps femoris tendon repair, each selected based on factors such as the extent of tendon injury, patient age, activity level, and underlying health conditions. Other surgical techniques commonly employed for quadriceps femoris tendon repair include: (1) Krackow suture technique: This method involves the use of locking loops to secure the tendon ends, providing robust fixation and promoting tendon healing. (2) Transosseous suture technique: In this approach, sutures are passed through bone tunnels created in the patella, facilitating strong tendon-to-bone fixation. (3) End-to-end repair: In cases of partial tendon tears or avulsion injuries, direct end-to-end repair may be feasible, promoting tendon healing and restoration of function. (4) Tendon graft augmentation: In instances of severe tendon damage or chronic ruptures, tendon grafts sourced from autografts or allografts may be utilized to reinforce the quadriceps femoris tendon and enhance biomechanical strength. Ultimately, the selection of the most appropriate surgical modality depends on careful consideration of individual patient factors and the specific characteristics of the tendon injury. Collaboration between orthopedic surgeons and rehabilitation specialists is essential to optimize surgical outcomes and facilitate patient recovery. Here, this article summarizes the surgical modalities associated with the quadriceps femoris tendon (Table 1).

**Table 1:** Risk factors and surgical treatment of quadriceps femoris tendon rupture reported in previous literature.

Age	Sex	Risk infactors	Surgical technique	Authors
44	Not mention	CKD	End-to-end suture using X-shaped stitches	(9)
46	Male	CKD	Primary repair	(10)
20	Female	CKD	Fix the tendon to the patella through nonabsorbable sutures	(17)
62	Male	None	Transosseous fixation	(15)
52	Male	None	Transosseous fixation	(20)
68	Female	None	Patellar resurfacing with an all-polyethylene 3-peg dome component	(16)
57	Female	CKD	Transosseous repair	(3)
26	Female	None	Modified Bunnel technique	(3)
43	Male	Fluoroquinolone-Associated	Primary repair	(14)
43	Male	Diabetic nephropathy	Transosseous fixation	(21)
34	Female	CKD	Transosseous fixation	(21)
54	Male	None	Transosseous fixation	(22)
55	Male	CKD	Modified knotless suture anchor and internal brace technique	(23)
56	Male	None	Transosseous fixation	(24)
45	Male	None	Primary repair	(25)
49	Male	None	Not mention	(26)
74	Male	Norfloxacin-associated	Not mention	(13)
40	Male	None	Transosseous fixation	(18)
60	Male	None	Krakow method	(27)
30	Male	CKD	Transosseous fixation	(8)

**Figure 5:**
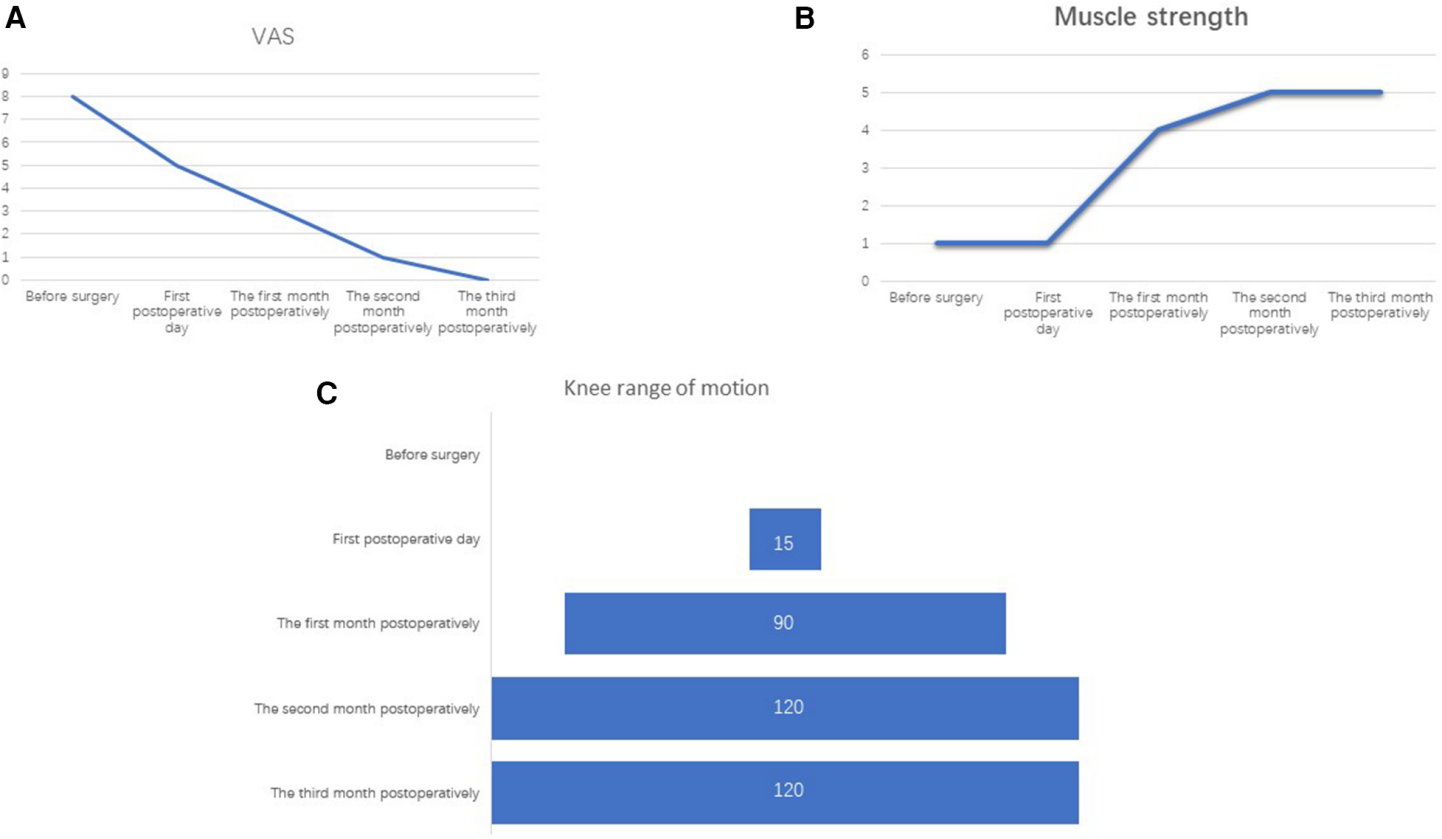
(**A**) The VAS pain scores, (**B**) muscle strength, and (**C**) knee range of motion for both lower limbs before surgery, on the first day after surgery, and at one month, two months, and three months post-surgery.

In the case reported in this article, the female patient had a preoperative VAS pain score of 8. Post-surgery, the pain significantly decreased, with the VAS score dropping to 5. During subsequent rehabilitation, the VAS pain scores further decreased to 3, 1, and 0 in the first, second, and third months post-surgery, respectively. The reduction in pain due to surgery facilitated the patient's rehabilitation exercises. Through ongoing rehabilitation education, the patient became more aware of the importance of rehabilitation and actively participated in training. The range of motion in both knees improved to 90° passive flexion by the end of the first month, 90° active flexion and 120° passive flexion by the end of the second month, and 110°–120° active flexion by the end of the third month (Figure 5), meeting the functional requirements for daily activities. In analyzing this case, we conclude that regardless of whether the patient undergoes surgery, the quadriceps tendon has the potential to heal. Without surgical treatment, pain symptoms can also be significantly reduced compared to preoperative levels. However, scar healing may lead to chronic pain over time. More importantly, non-surgical treatment involves many uncertainties regarding the healing of the quadriceps tendon, which can result in a less systematic and clear rehabilitation process. Therefore, through this case report, we hope to clarify that choosing surgical treatment for this type of condition can lead to better outcomes.

The analysis of the rare case of spontaneous bilateral quadriceps tendon rupture presented in this paper, along with relevant literature, highlights several key risk factors associated with this condition. Notably, a significant proportion of patients with quadriceps tendon rupture also have chronic kidney disease (CKD), underscoring the pivotal role of calcium and phosphorus metabolism in the pathogenesis of this condition. This observation further emphasizes the importance of systemic health in tendon integrity. Regarding surgical management, previous cases and literature reviews have demonstrated that tendon reconstruction for quadriceps tendon rupture typically involves suturing techniques, with or without bone tunnel drilling. In the case discussed here, a modified surgical approach was employed, involving absorbable sutures for tendon repair along with wire trapping to reduce tension. This innovative surgical technique not only addresses the tendon injury but also contributes to postoperative rehabilitation by minimizing tension on the repaired tendon. The favorable rehabilitation outcomes observed in this case highlight the effectiveness of the modified surgical approach in achieving satisfactory patient outcomes. By combining meticulous tendon repair with tension-reducing measures, this surgical method facilitates optimal healing and rehabilitation, ultimately leading to improved patient recovery and functional outcomes. This underscores the importance of individualized treatment strategies tailored to each patient's unique condition and needs in achieving successful outcomes in cases of quadriceps tendon rupture.

The treatment approach and principles for bilateral quadriceps tendon rupture vary depending on the patient's age and the presence of comorbidities. For elderly patients, surgery is essential once surgical contraindications are excluded. Prolonged bed rest in elderly patients can lead to severe complications such as aspiration pneumonia, pressure ulcers, and deep vein thrombosis, which can be fatal. Early surgery and prompt postoperative rehabilitation can reduce the occurrence of these complications, providing significant benefits for elderly patients. For patients with quadriceps tendon rupture who have other systemic diseases, a multidisciplinary approach is necessary. Combining surgical treatment with the management of underlying conditions can facilitate the growth of the quadriceps tendon post-surgery and reduce the likelihood of re-rupture. Controlling the source of the disease to some extent benefits postoperative recovery and overall patient outcomes.

In summary, for the rare condition of spontaneous bilateral quadriceps tendon rupture, early recognition and diagnosis are paramount for optimal patient outcomes. Clinicians should maintain a high level of suspicion for quadriceps tendon rupture, particularly after ruling out fractures during initial evaluation. Timely diagnosis enables prompt initiation of treatment, minimizing the interval between injury and surgery, which is crucial for achieving favorable results. Moreover, the quality and intensity of postoperative rehabilitation play a pivotal role in determining the long-term lower limb function of the patient. Early implementation of rehabilitation exercises, tailored to the individual patient's needs, promotes optimal healing and functional recovery. Ultimately, the combination of early and definitive diagnosis, individualized surgical ligament reconstruction, and timely initiation of appropriate postoperative rehabilitation exercises reflects the quality of comprehensive patient care. This integrated approach aims to optimize patient outcomes and enhance their overall quality of life following spontaneous bilateral quadriceps tendon rupture. In future research directions, we aim to uncover the pathological mechanisms of spontaneous bilateral quadriceps tendon rupture from a genetic perspective. Additionally, it remains to be determined whether there are clear differences in the prognosis between surgical and non-surgical treatments for this condition, necessitating further in-depth studies. Regarding improvements in treatment plans, continuous advancements in surgical techniques could enhance outcomes. This includes improving the tensile strength of sutures used for tendon repair, refining suturing methods to ensure appropriate tension post-repair, and exploring the feasibility of arthroscopic minimally invasive procedures. Such improvements in surgical techniques could significantly enhance the quality of postoperative rehabilitation. Therefore, we will continue to delve into these areas, striving to develop better treatment protocols and improve patient outcomes.

## Data Availability

The original contributions presented in the study are included in the article/**Supplementary Material**, further inquiries can be directed to the corresponding author.
