# Supplementary figures and images for: Rebuilding strength: surgical intervention and rehabilitation for bilateral spontaneous quadriceps tendon rupture—a case report
Source: Front Surg. 2024 Jul 18;11:1430774. doi: 10.3389/fsurg.2024.1430774 (PMC11291324; doi:10.3389/fsurg.2024.1430774)

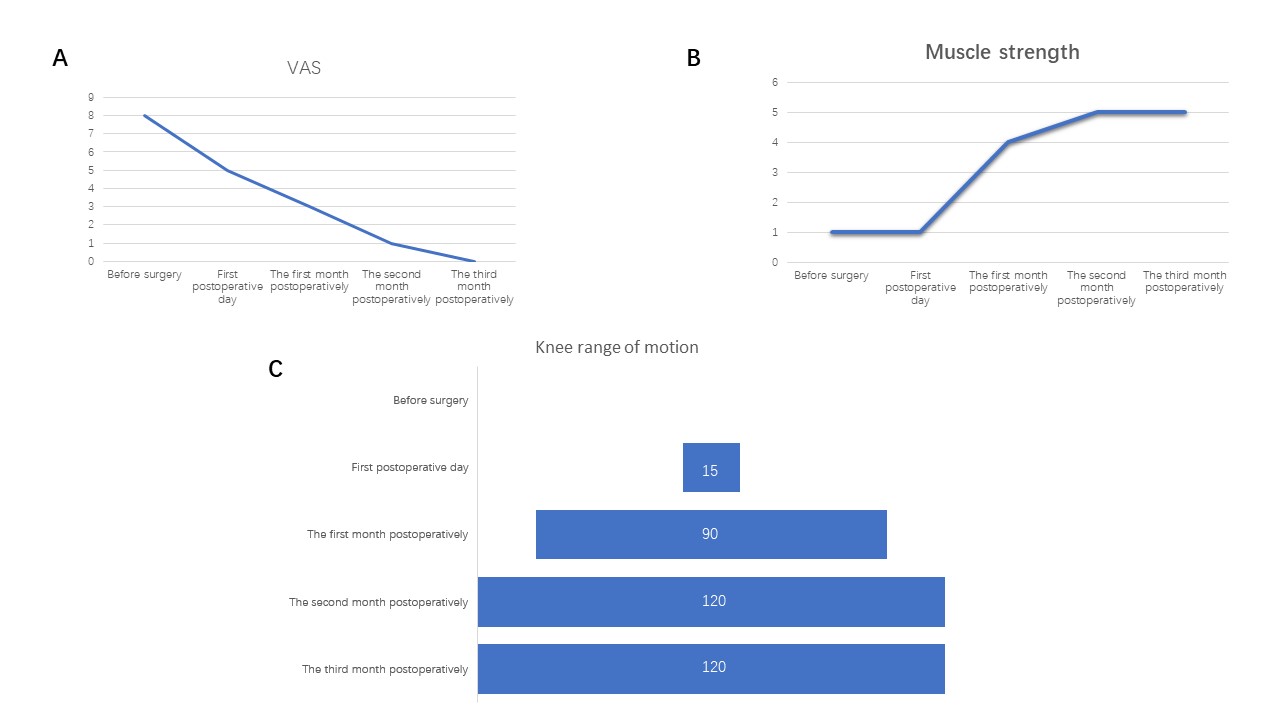

Supplement: Supplementary file 1 [file Image1.jpeg]
